# Initial Transcriptomic Response and Adaption of *Listeria monocytogenes* to Desiccation on Food Grade Stainless Steel

**DOI:** 10.3389/fmicb.2019.03132

**Published:** 2020-01-22

**Authors:** Martin Laage Kragh, Lisbeth Truelstrup Hansen

**Affiliations:** National Food Institute, Technical University of Denmark, Kongens Lyngby, Denmark

**Keywords:** stress response, RNA-seq, drying, osmotic stress, oxidative stress, sigma factors, motility, regulation

## Abstract

The foodborne pathogen *Listeria monocytogenes* survives exposure to a variety of stresses including desiccation in the food industry. Strand-specific RNA sequencing was applied to analyze changes in the transcriptomes of two strains of *L. monocytogenes* (Lm 568 and Lm 08-5578) during desiccation [15°C, 43% relative humidity (RH)] on food grade stainless steel surfaces over 48 h to simulate a weekend with no food production. Both strains showed similar survival during desiccation with a 1.8–2 Log CFU/cm^2^ reduction after 48 h. Analysis of differentially expressed (DE) genes (>twofold, adjusted *p*-value <0.05) revealed that the initial response to desiccation was established after 6 h and remained constant with few new genes being DE after 12, 24, and 48 h. A core of 81 up- and 73 down-regulated DE genes were identified as a shared, strain independent response to desiccation. Among common upregulated genes were energy and oxidative stress related genes e.g., *qoxABCD* (cytochrome aa_3_) *pdhABC* (pyruvate dehydrogenase complex) and *mntABCH* (manganese transporter). Common downregulated genes related to anaerobic growth, proteolysis and the two component systems *lmo1172*/*lmo1173* and *cheA/cheY*, which are involved in cold growth and flagellin production, respectively. Both strains upregulated additional genes involved in combatting oxidative stress and reactive oxygen species (ROS), including *sod* (superoxide dismutase), *kat* (catalase), *tpx* (thiol peroxidase) and several thioredoxins including *trxAB*, *lmo2390* and *lmo2830*. Osmotic stress related genes were also upregulated in both strains, including *gbuABC* (glycine betaine transporter) and several chaperones *clpC*, *cspA*, and *groE*. Significant strain differences were also detected with the food outbreak strain Lm 08-5578 differentially expressing 1.9 × more genes (726) compared to Lm 568 (410). Unique to Lm 08-5578 was a significant upregulation of the expression of the alternative transcription factor σ^B^ and its regulon. A number of long antisense transcripts (lasRNA) were upregulated during desiccation including *anti0605*, *anti0936*, *anti1846*, and *anti0777*, with the latter controlling flagellum biosynthesis and possibly the downregulation of motility genes observed in both strains. This exploration of the transcriptomes of desiccated *L. monocytogenes* provides further understanding of how this bacterium encounters and survives the stress faced when exposed to dry conditions in the food industry.

## Introduction

*Listeria monocytogenes* is an important Gram-positive bacterial pathogen responsible for 46% (225) of the deaths reported to be caused by food-borne and zoonotic pathogens in 18 European Union (EU) countries in 2017. Outbreaks of listeriosis are characterized by a high case fatality (∼10–30%), making the observed rise in listeriosis cases in the EU since 2008 (EFSA and ECDC, 2018) a serious concern for the health of vulnerable population groups and necessitating research into the control of the organism.

The ability of *L. monocytogenes* to survive and persist in food production environments for several years has been reported in numerous studies (Miettinen et al., 1999; Wulff et al., 2006; Orsi et al., 2008). These house-strains of *L. monocytogenes* have been associated with listeriosis outbreaks and product recalls leading to illness and considerable economic losses (Larsen et al., 2014; Thomas et al., 2015). As *L. monocytogenes* is ubiquitously distributed in the environment (e.g., soil, water, plants, animals, human), entry into food productions facilities can occur through several contamination routes including raw materials, personnel and equipment as reviewed by NicAogáin and O’Byrne (2016). The contamination of food contact surfaces with *L. monocytogenes* increases the risk of cross-contamination of foods with epidemiological data pointing to *L. monocytogenes* derived from the food processing environment being the main cause for contamination of ready-to-eat foods (Midelet et al., 2006; Rodríguez and McLandsborough, 2007; EFSA BIOHAZ Panel (EFSA Panel on Biological Hazards) et al., 2018).

*Listeria monocytogenes* faces a variety of adverse conditions in the food processing environments including low pH, oxidative, osmotic pressure changes, desiccation, low and high temperature, UV light, and exposure to various disinfectants and sanitizers (Bucur et al., 2018). The ability to adhere to and stay viable on stainless steel surfaces for at least 3 months at low relative humidity (RH) indicates a role of desiccation tolerance in persistence of *L. monocytogenes* (Norwood and Gilmour, 1999; Vogel et al., 2010). Moisture and niches with water in food production facilities should be minimized to prevent microbial growth and formation of biofilms. *L. monocytogenes* can survive air-drying whether in a dry processing environment or exposure to air-dehumidification operations following wet-cleaning (Esbelin et al., 2018). Drying and dry environments with low RH exposes *L. monocytogenes* to a gas phase with a water activity (*a*_w_) that is lower than inside the cell leading to removal of water from the cells and induction of a so-called desiccation or matric stress (Potts, 1994). Matric stress is characterized by elements of both osmotic and oxidative stress due to the disappearance of water and increasing cellular exposure to the atmosphere (Billi and Potts, 2002).

The majority of our knowledge about the desiccation stress response of *L. monocytogenes* is based on osmotic stress studies, which points to the need for dedicated studies to investigate the specific mechanisms involved in the desiccation stress response of the bacterium (Burgess et al., 2016; Esbelin et al., 2018). Phenotypic and genotypic studies have attempted, without reaching fully conclusive observations, to relate specific genotypes to increased or decreased resistance to stresses such as cold, acid, salt, and desiccation (Hingston et al., 2017b; Pasquali et al., 2018; Zhang et al., 2018). The origin of strains was observed to be less important for desiccation resistance in the studies of Faleiro et al. (2003) and Zoz et al. (2017), while Vialette et al. (2003) could differentiate strains from seafood and clinical sources based on their osmotic stress tolerance with the latter being more osmotolerant. Presence of a five gene stress survival islet 1 (SSI-1) was reported to be important for acid, salt and cold growth in *L. monocytogenes* (Cotter et al., 2005; Ryan et al., 2010). However, recent studies found no significant differences in cold, acid, salt, and desiccation stress tolerances between strains with and without SSI-1 (Arguedas-Villa et al., 2014; Hingston et al., 2017b), indicating the SSI-1 cannot be used as a determinant of stress tolerance. Similar to other bacteria, endogenous presence, cellular uptake and/or accumulation of osmoprotectants such as carnitine, glycine betaine, trehalose, and proline have been shown to exert positive effects on desiccation tolerance of *L. monocytogenes* (Cayley et al., 1992; Beumer et al., 1994; Ells and Hansen, 2011; Huang et al., 2015). A transposon mutant study identified 23 desiccation tolerant and sensitive mutants with interruption in genes involved in lipid biosynthesis, energy production, membrane transport, secreted internalins or oxidative damage control (Hingston et al., 2015). The study additionally identified seven immotile desiccation tolerant mutants with insertions in motility related genes. To overcome the gap in our knowledge of changes in *L. monocytogenes* cells being dried, application of the RNA-seq technology enables a comprehensive study of the global cellular response. The present study is to the best of our knowledge the first study to apply a RNA-seq based whole transcriptomic analysis on *L. monocytogenes* being desiccated under industrially relevant conditions.

The objective of this study was to explore the initial transcriptomic response and adaptation of *L. monocytogenes* during the initial 6–48 h of desiccation on stainless steel as previous desiccation experiments have shown that the biggest loss in viability happens within the first 2 days (Hingston et al., 2013). To accomplish this, RNA sequencing of the transcriptomes was conducted after 0 (control), 6, 12, 24, and 48 h of the onset of desiccation. Two *L. monocytogenes* strains [a food (Lm 568) and a food outbreak related clinical strain (Lm 08-5578)], RH (43%), temperature (15°C), desiccation period (48 h), and surface (food grade stainless steel) were all chosen in order to mimic conditions faced during the weekend shut-down of a food processing plant to make potential findings relevant in development of new mitigation strategies to prevent *L. monocytogenes* contamination of food products during processing.

## Materials and Methods

### Strains and Culture Conditions

The food strain *L. monocytogenes* 568 (Serotype 1/2a, Kalmokoff et al., 2001), originally isolated from a shrimp processing plant and the food outbreak related clinical strain *L. monocytogenes* 08-5578 (Serotype 1/2a, Gilmour et al., 2010), originally isolated from a human blood sample in the 2008 Canadian foodborne listeriosis outbreak, were selected. Two serotype 1/2a strains were used in the study to highlight potential differences in the transcriptomic response among two strains belonging to the same serotype. Strains were stored in a 20% (v/v) glycerol peptone medium (TS/80, Technical Service Consultants Ltd., Heywood, United Kingdom) at −80°C. Strains were revitalized and routinely cultured at room temperature (20–22°C) on Tryptic Soy Agar plates (TSA), composed of 30 g/L TSB (TSB, Merck, Darmstadt, Germany) and 15 g/L agar (Sigma-Aldrich, St. Louis, MO, United States).

### Desiccation of *L. monocytogenes* on Stainless Steel

Prior to desiccation survival experiments, both strains were pre-cultured in 50 ml Tryptic Soy Broth (TSB-glu) [TSB supplemented with 1% (w/v) glucose (Thermo Fisher Scientific, Loughborough, United Kingdom)] for 48 h at 15°C. Inoculation of each pre-culture was done using several fresh colonies from TSA plates in order to avoid introduction of undesired variability between biological replicates. The stainless steel (SS, food grade AISI 316, type 4 finish, thickness 1 mm) plates of 8 × 2 cm were cleaned and autoclaved as described by Hingston et al. (2013) and placed horizontally on a stainless steel rack in a biosafety cabinet before inoculation. Pre-cultures were harvested by centrifugation at 2300 × *g*, for 5 min and resuspended in TSB-glu to OD_600__nm_ of 2 (NP80 NanoPhotometer, Implen, Westlake Village, CA, United States). The final cell concentrations were approximately 2 × 10^9^ CFU/ml and 640 μl of this culture was spread on one side of the sterile stainless steel plates resulting in a concentration of 10^8^ CFU/cm^2^. Twenty SS plates were inoculated for each strain. A previous study had shown the desiccation survival kinetics of *L. monocytogenes* to be independent of initial contamination levels (Hingston et al., 2013) and thus a high cell concentration was chosen in order to enable the harvest of sufficient RNA for RNA-seq. The rack containing a total of 40 SS plates were quickly transferred to a HPP 110 Memmert Constant Climate Chamber (Memmert GmbH + Co.KG, Schwabach, Germany) with the RH and temperature kept constant at 43% and 15°C, respectively. To help stabilizing the RH by absorbing the water evaporating from the cultures, 1000 g of silica gel was filled into petri dishes and placed on a SS rack located approximately 30 cm above the SS plates. The RH and temperature in the climate chamber were logged throughout the desiccation experiments, during which RH was continuously maintained at 43% by introducing moisture from an external source (container with water). Three biological independent replicate desiccation experiments were performed for both *L. monocytogenes* strains, where triplicate samples for enumeration of survivors and duplicate samples for RNA isolation were obtained after desiccation for 0, 6, 12, 24, and 48 h. Control samples (0 h, i.e., no desiccation) consisted of freshly inoculated SS plates that were immediately sampled.

### Enumeration of Desiccation Survivors

Numbers of surviving *L. monocytogenes* were enumerated in triplicates at all time points for each strain. Three SS plates from each strain were removed from the climate chamber and placed in individual tubes with 50 ml peptone saline [PS, 0.1% Peptone (Oxoid, Hampshire, United Kingdom), 0.85 g NaCl (Merck)]. Adhering cells were released from the SS plates by sonication for 5 min with 50/60 kHz in a sonication bath (Elmasonic S 120, Thermo Fisher Scientific) followed by vortexing for 20 s. This protocol for release of bacteria is adapted from the method originally developed by Leriche and Carpentier (1995). Samples were then serially diluted in PS and appropriate dilutions were spot plated (three drops of 20 μL of each suitable dilution) on TSA plates. Colonies were enumerated after incubation for 48 h at room temperature and expressed as Log CFU/cm^2^.

### RNA Isolation and Sequencing

At each time point two SS plates from each strain were placed directly in individual centrifuge tubes containing 50 ml of an ice cold stop solution [90% PS, 9% ethanol (96% v/v), WVR] and 1% Phenol:Chloroform:Isoamyl Alcohol (25:24:1) (Thermo Fisher Scientific) (Hingston et al., 2017a). The tubes were vortexed briefly before adhering cells were released from the SS plates by sonication for 5 min with 50/60 kHz in a sonication bath (Elmasonic S 120) filled with ice water followed by vortexing for 10 s. SS plates were removed before pelleting the cells by centrifugation for 10 min at 7.400 × *g* at 0°C. The supernatant was carefully removed followed by resuspension of the pellet in the remaining supernatant, transfer to microcentrifuge tubes and centrifugation at 9.900 × *g* for 1 min. Harvested cells from the two replicate SS plates, for each time point and strain, were pooled into one microcentrifuge tube and stored at −80°C for less than 1 week before RNA isolation. Total RNA was isolated and purified using the RNeasy PowerMicrobiome kit (Qiagen, Hilden, Germany) according to the manufacturer’s instructions with RNA eluted in 60 μL RNase-free water. An extra optional DNAse treatment using the DNAse MAX Kit (Qiagen) was done after preliminary tests proved the on-column DNase treatment to be insufficient. RNA was quantified using Qubit 3.0 (Invitrogen, Carlsbad, CA, United States) with the Qubit RNA HS Assay Kit (Invitrogen). The RNA integrity numbers (RINs) were assessed using the 2100 Bioanalyzer (Agilent, Santa Clara, CA, United States) with the Agilent RNA 6000 Nano Chip kit. Samples containing 40 μL total RNA with a RIN between 8.7–10 and RNA concentrations >180 ng/μl were sent to Eurofins Genomics (Ebersberg, Munich, Germany) for rRNA-depleted Illumina TruSeq RNA library preparation and TruSeq stranded total RNA 100 bp paired-end (PE) sequencing on the Illumina HiSeq 2500 platform.

### RNA-seq Data Analysis

RNA sequencing results from the three independent biological replicate experiments for each strain were named according to the experiment (H, N, P), strain (Lm 568 or Lm 08-5578) and duration of desiccation (0, 6, 12, 24, 48 h). Sequencing quality was evaluated using FastQC (Andrews, 2010). Adapter sequences and low-quality base pairs were removed using CLC Genomics 12 (Qiagen Bioinformatics, Aarhus, Denmark) with a custom made adapter list based on QC reports from FastQC. After the removal of low-quality reads, 19.1–39.6 million reads remained for each sample with an average of 26 million reads. Reads from Lm 568 and Lm 08-5578 were mapped to the complete sequenced genomes of *L. monocytogenes* EGD-e (NCBI RefSeq NC_003210.1) and *L. monocytogenes* 08-5578 (NCBI RefSeq CP_001602 and CP001603) using CLC Genomics 12. The mapping efficiency for individual samples ranged from 88.89 to 98.19% and 95.38 to 97.72% for Lm 568 and Lm 08-5578, respectively. Ten samples (five from each strain) with low mapping % [all from the same biological experiment (N)] were identified as outliers based on a principal component analysis (PCA) and omitted from further downstream analyses. Disregarding the outliers, the mapping efficiency for the reads in individual samples now ranged from 97.20 to 98.19% and 96.67 to 97.72% for Lm 568 and Lm 08-5578, respectively. Counting of reads and differential gene expression (DGE) analysis were done within CLC Genomics 12 with the trimmed mean of M values (TMM) normalization method (Qiagen Bioinformatics, 2019). TMM is the normalization method used in the widely used Bioconductor R-package EdgeR for DGE analysis (Robinson et al., 2009). PCA was conducted in CLC Genomics 12 using the TMM normalized counts to identify and remove outliers in the dataset. DGE results were reported as significant if Log_2_ fold changes (LFC) were >1 (i.e., >twofold change) with an adjusted *p*-value < 0.05. The adjusted *p*-value was calculated based on the Benjamini-Hochberg (BH) adjustment method (Benjamini and Hochberg, 1995) using CLC Genomics 12. DGE was analyzed for each strain comparing the control (0 h, *n* = 2) to each time point (*n* = 2). Based on the observed similarity in the transcriptomes of all desiccated samples, the DGE analysis was repeated for each strain using the two biological controls (0 h, *n* = 2) in a comparison to expression in the eight desiccated samples (i.e., pooling the results from 6, 12, 24, and 48 h, *n* = 8).

### Categorization and Enrichment Analysis of Differentially Expressed Genes

To explore and analyze the functional and biological roles of the differentially expressed (DE) genes during desiccation, the BioCyc database^1^ were used to compare EGD-e genes to *L. monocytogenes* 10403S genes for which the BioCyc database contains manually curated data with transcription regulation and regulon information (Orsi et al., 2015). Corresponding 10403S genes were used to map identified DE genes on the Cellular Overview and Omics Dashboard to group the DE genes based on biological subsystems and processes (Caspi et al., 2016). Overrepresentation tests of DE genes for specific regulons were done using the BioCyc Smarttables (Fisher’s exact test, *p* < 0.05), while overrepresentation tests of biological processes were done using PANTHER Overrepresentation Test^2^ using the GO Biological Process as annotation data set (Fisher’s exact test, *p* < 0.05) (Mi et al., 2019).

### Reverse Transcription Quantitative PCR Validation of RNA-seq Data

The RNA-seq results were validated using reverse transcription quantitative PCR (RT-qPCR) amplification of four genes: (1) *qoxB* which exhibited >fourfold higher expression during desiccation, (2) *lmo1634* which consistently exhibited >fourfold lower expression during desiccation, (3) *inlH* which exhibited >fourfold higher expression during desiccation in *Lm 08-5578*, but a non-significant downregulation in *Lm 568* at all four time points during desiccation, and (4) *lmo1524* (*recJ*), which was chosen as reference gene based on its stable expression across all five time points (Supplementary Table S1). Up to 1 μg of RNA from each of 20 samples was reverse transcribed using the QuantiTect Reverse Transcription Kit (Qiagen) according to the manufacturer’s protocol. Primers were designed using CLC Genomics 12 (Table 1) using the complete sequenced genome of *L. monocytogenes* EGD-e (NCBI RefSeq NC_003210.1). The qPCR was performed with technical duplicates in optical tubes and caps (Agilent Technologies, Santa Clara, CA, United States) in a Stratagene Mx3000p qPCR System (Agilent Technologies) with an initial 3 min denaturation step at 95°C followed by 40 cycles of repeated denaturation at 95°C for 10 s, annealing at 55°C for 20 s and elongation at 72°C for 20 s. The 2^–ΔΔCT^ method was used to determine the relative expression levels of *qoxB*, *lmo1634*, and *inlH*, with *recJ* used as the reference gene (Livak and Schmittgen, 2001).

**TABLE 1:** Primers used for RT-qPCR validation of RNA-seq data.

**Gene**	**Name**	**Primer sequence (5′–3′)**	**Source**
*qoxB*	qoxB_F	**Fw-**ACAGCATTCTTCACACTCACGA	This study
	qoxB_R	**Rv-**GCCCCCAAACCCAGAACAA	
*lmo1634*	lmo1634_F	**Fw**-GCGCGCGAAAAAATGCATAA	This study
	lmo1634_R	**Rv-**TGTGTGCCAAGCTGTGGT	
*inlH*	inlH_F	**Fw-**ATGGGATTT TGCGACAGGG	This study
	inlH_R	**Rv-**CGGGAT TCGGGT TGTCATT	
*recJ*	recJ_F	**Fw-**CTCGACCGGCAATTGTGTTG	Hingston et al., 2017a
	recJ_R	**Rv**-GTCCACACTTCGACCAGACC	

### Statistical Analyses

Results from the desiccation experiments were log_10_ transformed (Log CFU/cm^2^) and expressed as (ΔLog CFU/cm^2^) by subtracting the viable count before desiccation (0 h) from the viable count at each desiccation time point. Results were expressed as means ± standard deviation for each strain from three biologically independent trials with triplicate samples for each time point (*n* = 9). *T*-tests at a significance level of 5% were performed when comparing the number of survivors of Lm 568 and Lm 08-5578 at each time point during desiccation. Survival kinetics were modeled for each strain using the Weibull model (Mafart et al., 2001). The Weibull model parameters (delta and P) were obtained using the Microsoft Excel^®^ Add-in software, GInaFIT (version 1.6) available at KULeuven/BioTec^3^ and developed by Geeraerd et al. (2005). The model parameters of each strain were compared using *t*-tests. Statistical analysis of RNA-seq data were done as described above.

## Results and Discussion

### Survival of *L. monocytogenes* During Desiccation on Stainless Steel

**FIGURE 1:**
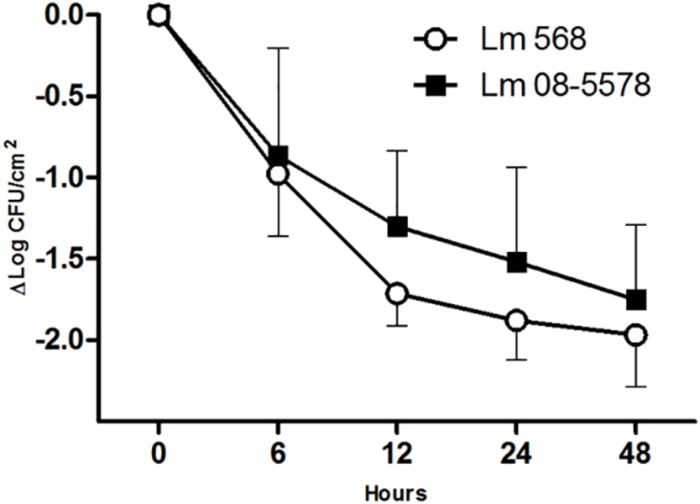
Desiccation survival of *L. monocytogenes* 568 and 08-5578 on a food grade stainless steel surface. Cells were pre-cultured at 15°C in TSB-glu and re-suspended in TSB-glu before being desiccated (43% RH, 15°C) for 48 h with a starting concentration of 8 × 10^7^ CFU/cm^2^. Cells were enumerated by plate counts. Symbols are averages of three biological independent experiments with triplicates (*n* = 9). Error bars indicate standard deviation.

Desiccation of *L. monocytogenes* on stainless steel for 48 h at a RH of 43% at 15°C resulted in overall losses of culturable cells for Lm 568 and Lm 08-5578 of 1.97 ± 0.32 and 1.81 ± 0.46 Log CFU/cm^2^, respectively (Figure 1). Both strains saw the biggest losses in viability during the first 6 to 12 h after which inactivation rate declined. This is in line with previous findings for Lm 568 where the biggest loss in viability (1.5–2.0 Log CFU/cm^2^) occurred before the first sampling point (48 or 24 h), after which the concentration of surviving cell only slowly declined (Hingston et al., 2013, 2015). Lm 08-5578 exhibited a slightly but insignificantly (*p* > 0.05) better desiccation tolerance compared to Lm 568. Also, there were no significant (*p* > 0.05) differences between the model parameters when the survival kinetics of the two strains were compared using the Weilbull model (Mafart et al., 2001) (data not shown). Lm 08-5578 has previously been shown to display increased desiccation tolerance compared to Lm 568 in desiccation trials at 23% RH and 15°C (Piercey et al., 2017), indicating that Lm 08-5578 may have an advantage at a lower RH.

### Analysis of Sample mRNA Transcriptomes

A total of 17–34 million PE mRNA reads per sample were successfully matched to an *L. monocytogenes* EDG-e open reading frame (ORF). The number of counts to each EGD-e ORF ranged from none (0) to 279.000 and 0 to 328.000 for Lm 568 and Lm 08-5578, respectively, and for both strains there were 176 (5.8%) and 150 (4.9%) ORFs, respectively, for which there were no counts in any of the samples. The obtained RNA-seq results were confirmed using RT-qPCR to assess the gene expression of three genes (*qoxB*, *lmo1634*, and *inlH*). The RT-qPCR results matched the RNA-seq results resulting in a positive correlation (*R*^2^ = 0.91, *y* = 1.05*x* − 0.23) being observed between the mRNA expression levels detected by RNA-seq and RT-qPCR (Supplementary Figure S1).

### Changes in the mRNA Transcriptome of *L. monocytogenes* Exposed to Desiccation Stress

**FIGURE 2:**
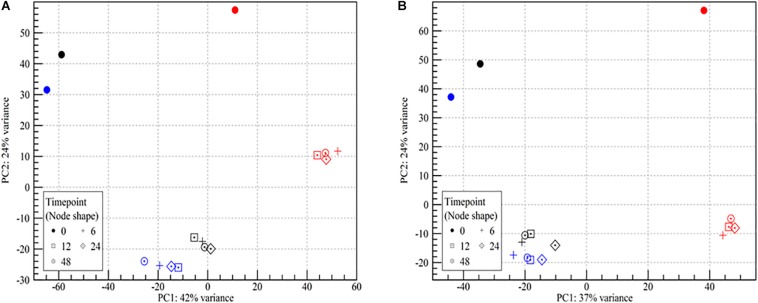
Principle component analysis plot of RNA-seq read counts in samples of wet and desiccating *L. monocytogenes* cells on a stainless steel surface. **(A)** Lm 568, **(B)** Lm 08-5578. Symbol shape refers to sampling time (number of hours desiccated 43% RH and 15°C): 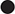 (wet, control at 0 h), + (6 h), ⊡ (12 h), ♢⋅ (24 h), ⊙ (48 h). Symbol colors refer to the three biological independent replicate experiments. All samples from the experiment with red symbols were excluded from further analyses due to an abundance of outlier data points.

The mRNA transcriptomes showed pronounced changes as the cells went from being wet to dry (PC2, 24% of variance, Figure 2). Once the desiccation stress began, differences among the desiccated samples became insignificant regardless of the duration of the desiccation (PC1, Figure 2). While samples from both strains showed highly similar trends in two of the three biological replicates, results for both strains in the third biological replicate (N) deviated at all time points (Figure 2). As this replicate was identified as an outlier it was chosen to omit it from further analyses. The lower mapping % for replicate N points to a problem with the sequencing, as great care was taken to standardize all experimental conditions. It should be noted that enumeration of the desiccation survivors showed no significant (*p* > 0.05) differences among the three biological replicates (Figure 1). This issue underlines the importance of using biological replicates in RNA-seq, which we in this study also augmented by studying the desiccation related transcriptomes of two different strains.

**FIGURE 3:**
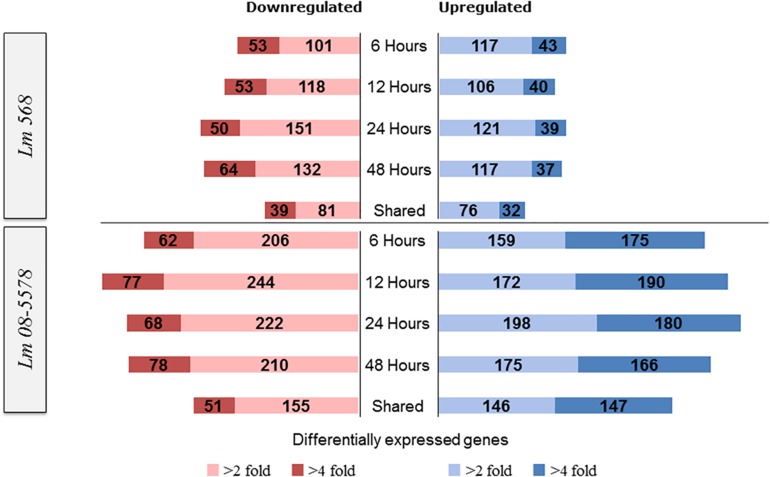
Numbers of *L. monocytogenes* 568 and 08-5578 differentially expressed (DE) genes in response to desiccation stress (desiccated samples relative to wet control at 0 h). Differential expressed genes were reported significantly DE with fold changes >2 or 4 and an adjusted *p-*value < 0.05. The sampling times correspond to the survivor curve (Figure 1). Shared corresponds to the number of DE genes that were shared among all desiccated samples.

Statistical analyses (Pearson correlations) of the transcriptomes confirmed the similarities among all desiccated samples with correlation coefficients ranging from 0.97 to 0.99 (Supplementary Figure S2). In comparison, correlation coefficients between the wet controls and desiccated samples ranged from 0.71 to 0.78. This similarity among the desiccated samples was also evident when looking at the number of DE genes throughout the desiccation period (Figure 3).

For Lm 568, the number of significantly upregulated genes at each desiccation time point varied from 146 to 160 with 108 genes being shared among the four desiccation samples (6–12–24–48 h) and thus consistently upregulated (Figure 3). For Lm 08-5578, numbers ranged from 334 to 378 with 293 genes upregulated in all four desiccation samples (shared). In both strains, this shared desiccation response constituted most of the DE genes corresponding to 63.0–72.9% and 73.1–82.9% of the DE genes at the four time points for Lm 568 and Lm 08-5578, respectively. Overall 1.2× more DE genes were found to be downregulated than upregulated in Lm 568, while 1.2× more genes were upregulated during desiccation of Lm 08-5578. Additionally, the number of significantly up- and downregulated genes in Lm 08-5578 was nearly twice (1.9×) the total number of DE genes in Lm 568, indicating the food outbreak related clinical Lm 08-578 isolate harbored a comprehensive response to desiccation stress. This observation was also evident in the number of highly DE genes (>fourfold change) where the number and proportion of upregulated genes were higher in Lm 08-5578 than in Lm 568 (Figure 3). The use of reference genomes when mapping reads can introduce differences, but mapping Lm 08-5578 to its own genome instead of Lm EGD-e only changed the number of DE genes by a mean of 11 genes at each time point with the same number (499) of DE genes being shared among all desiccated samples. Together these observations indicates a larger response in Lm 08-5578 both in terms of the number of DE genes and >fourfold DE genes during desiccation.

**FIGURE 4:**
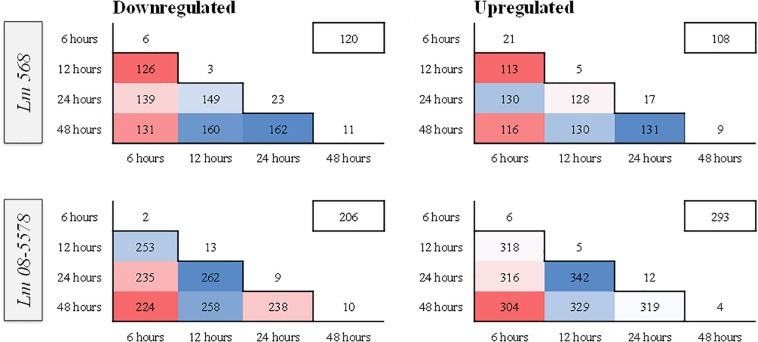
Heatmaps showing similarity in numbers of differentially expressed (DE) genes between different sampling times in *L. monocytogenes* 568 and 08-5578. Numbers inside boxes corresponds to number of DE genes (LFC > 1, adjusted *p-*value < 0.05) shared between desiccation times. Numbers outside the pyramid are the number of unique down- or upregulated at each desiccation time. The number in the boxes on the upper right represents the number of DE genes shared among all desiccated samples for each strain.

Figure 4 shows the stable number of DE genes shared between two time points as well as the low number of unique DE genes present for each desiccation time. This points to the transcriptomic response to desiccation on stainless steel over 48 h being a stable core response without significant changes in gene expression occurring after the initial response (6 h). In contrast (Wen et al., 2011) identified transitions in gene expression when studying the transcriptomes during long-term-survival of *L. monocytogenes* in liquid culture media. They did, however, report that the transcriptomes in the final two samples (168 and 336 h), where the reduced cell numbers had stabilized, were highly similar.

### Strain Independent Core Desiccation Stress Response in *L. monocytogenes*

The core transcriptomic response to desiccation in both *L. monocytogenes* strains was identified using a strict statistical filter that only picked genes found to be DE in all desiccated samples from both strains. The resulting core response to desiccation stress was composed of a total of 154 genes, where 81 genes (Table 2) and 73 genes (Table 3) were significantly up- or downregulated, respectively, in both strains. Using intensity based coloring of the DGE it is noticeable how the LFC is practically identical throughout the 48-h desiccation period. Additionally, it is evident that most genes with high LFCs were induced in both strains.

**TABLE 2:** Core set of genes upregulated (>twofold) in both strains in response to desiccation.

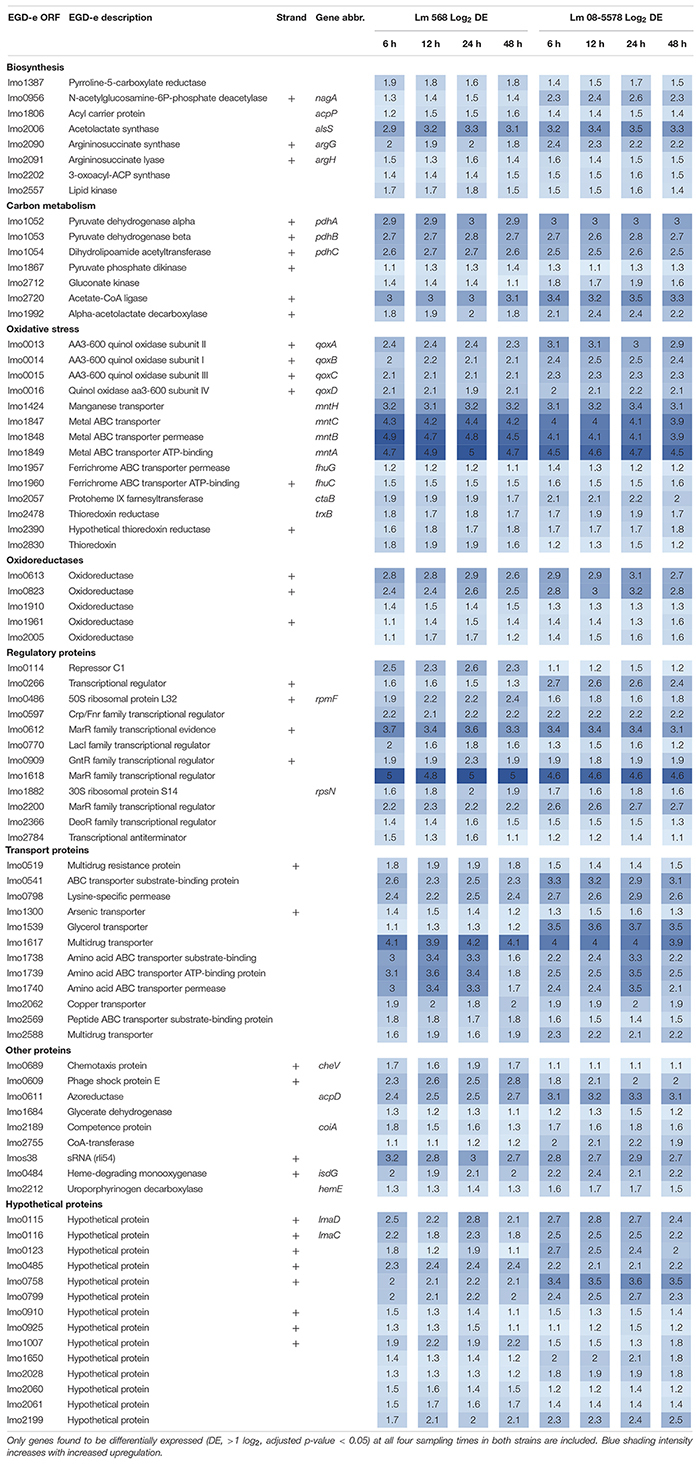

**TABLE 3:** Core set of genes downregulated (<twofold) in both strains in response to desiccation.

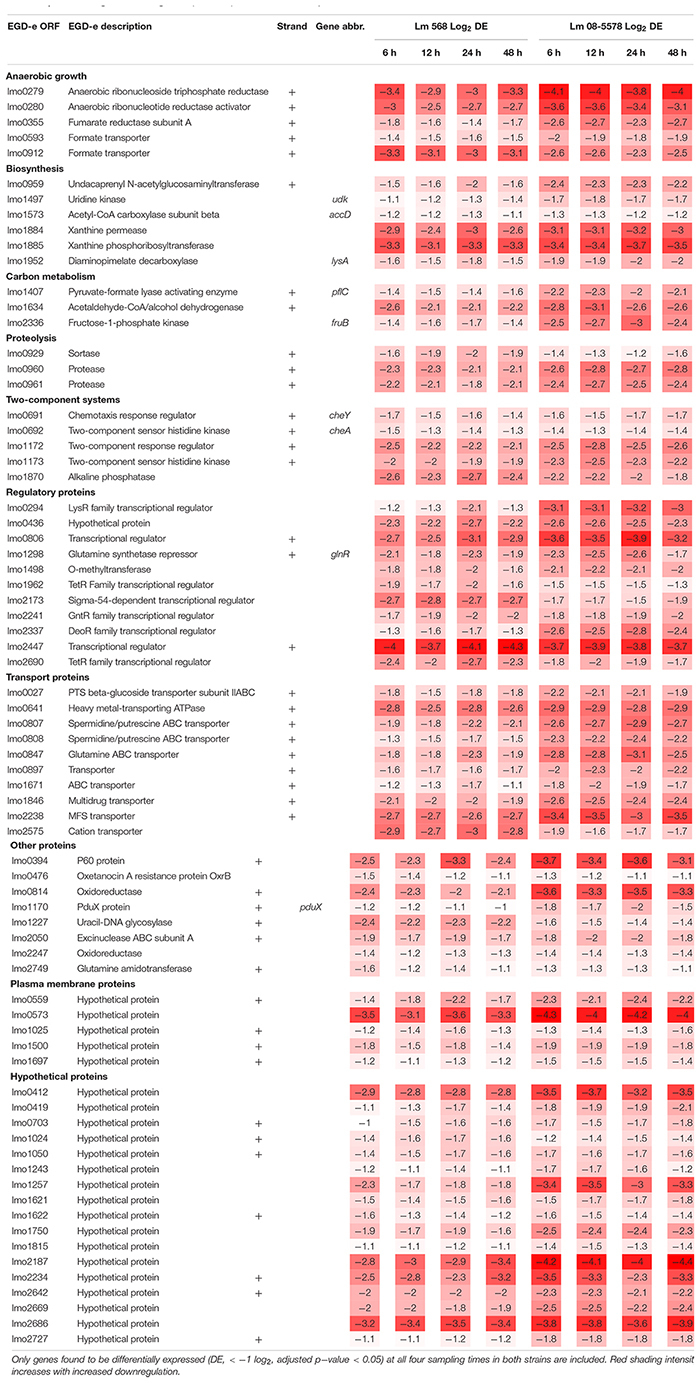

As evident from the list of upregulated core genes (Table 2), it contains genes involved in several response mechanisms which have previously been related to oxidative, salt and acid shock stress. Among upregulated genes were the carbon-metabolism related and σ^B^-regulated *pdh* operon, encoding the pyruvate dehydrogenase complex, where *pdhA*, *pdhB*, and *pdhC* have previously been observed to be induced by salt and acid shock (Duché et al., 2002; Satorhelyi, 2005). The last gene in the salt shock induced operon, *pdhD*, was upregulated at all times, however, this was not significant (*p* > 0.05) after desiccation for 6 and 24 h in Lm 568 and Lm 08-5578, respectively. The σ^B^-regulated quinol oxidase operon (*qoxABCD*), which encodes cytochrome aa_3_ and is involved in oxidative stress response and required for aerobic growth (Corbett et al., 2017; Liu et al., 2017a), was part of the shared core response to desiccation with upregulation of all four genes (Table 2). The importance of oxidative stress is supported by the observed upregulation of *ctaB*, which is needed for biosynthesis of the prosthetic group in cytochrome aa_3_ (Svensson et al., 1993). Moreover four metal ABC transporters *mntACBH* were highly upregulated (>16 fold, Table 2) and together these form an ATP-driven manganese transport system (Horsburgh et al., 2002) that strongly supports an oxidative stress response being an essential part of the response to desiccation. In addition, thioredoxin reductase *trxB*, regulated by the peroxide operon regulator PerR and regarded as part of oxidative stress responses (Rea et al., 2005), was also upregulated together with two other thioredoxin reductases (*lmo2390* and *lmo2830*) (Table 2). The most strongly upregulated gene (>24 fold) was in both strains a transcription regulator of the MarR family (*lmo1618*) previously related to alkaline and oxidative stress (Rea et al., 2004). Additionally the *lmo1738*-*1740* operon encoding glutamine transporters was highly upregulated similarly to what was reported for acid shock, and may likely be linked to the essential role that the glutamate decarboxylase system (GAD) plays in intercellular pH homeostasis (Satorhelyi, 2005; Feehily and Karatzas, 2013). Five oxidoreductases coding genes were upregulated (Table 2). Oxidoreductases have been linked to acid resistance in *L. monocytogenes* working to deacidify the cytoplasm to keep cellular pH homeostasis (Phan-Thanh and Jänsch, 2005). In desiccated *Salmonella enterica* oxidoreductases were also observed to be among the more upregulated group of genes (Gruzdev et al., 2012). Overall, overrepresentation tests of biological processes showed the upregulated core response to be significantly enriched in genes involved in the regulation of transcription (GO:0006355) and the respiratory electron transport chain (GO:0022904).

Seventy-three genes were downregulated as both strains responded to desiccation (Table 3). Among these genes were the two-component systems, *cheA*/*cheY* and *lmo1172*/*lmo1173*, where the former is the chemotaxis two-component system regulating flagella expression in response to environmental signals with both Δ*cheA* and Δ*cheY* mutants showing reduced amounts of flagellin and flagella (Flanary et al., 1999). In contrast to this observation, chemotaxis *cheV* were upregulated in all desiccated samples (Table 2). The *lmo1172*/*1173* operon is positively regulated by σ^L^ during cold growth and deletion of this systems led to poor growth at 4°C (Chan et al., 2008; Mattila et al., 2012). While genes within the respiratory electron transport chain were upregulated during desiccation, there was a downregulation of several genes related to anaerobic growth. Two anaerobic reductases (*lmo0279*-*80*) were among the most downregulated genes (LFC 2.5-4.1, Table 3). Two formate transporters (*lmo0593*, *lmo0912*) and a fumarate reductase (*lmo0355*) were downregulated explained by the evidence of formate being a metabolic end product only seen during anaerobic growth (Romick et al., 1996). In line with the upregulation during desiccation of glutamine transporters, *glnR* (*lmo1298*), a glutamine synthase repressor (Vivant et al., 2017) was downregulated. The most downregulated gene (>13 fold) during desiccation were the predicted transcriptional regulator *lmo2447* (Table 3). Overrepresentation tests of biological processes showed the downregulated core response to be significantly enriched in regulation of transmembrane transport (GO:0055085) and primary metabolic process (GO:0044238).

### Strain Dependent Desiccation Stress Responses in *L. monocytogenes*

**FIGURE 5:**
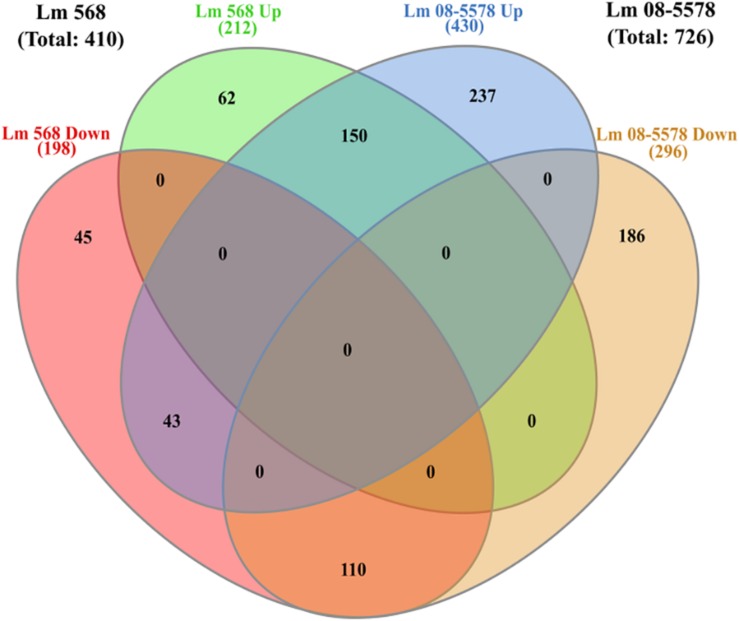
Venn diagram of differential expressed genes in response to desiccation in *Listeria monocytogenes* 568 and 08-5578. Number of DE genes (LFC > 1, adjusted *p-*value < 0.05) for each strain are based on RNAseq analysis after grouping of desiccated samples (6, 12, 24, and 48 h) into one group of desiccated samples (*n* = 8) compared to wet control (*n* = 2). Circles indicate common and unique DE up- and downregulated genes. Numbers in parenthesis refers to strain totals of each group.

As only minimal changes characterized the transcription patterns during the course of 48-h desiccation period, it was decided to pool all desiccation transcriptomes from the four desiccation sampling points (6–12–24–48 h, *n* = 8 for each strain) and redo DE analysis. This resulted in 410 genes (Supplementary Table S2) being DE by Lm 568 in response to desiccation, in contrast to the 314–362 DE genes that were detected when individual desiccation times were compared to the wet control (Figure 3). In Lm 08-5578 the same analysis identified 726 DE genes (Supplementary Table S2) during desiccation as opposed to 602–683 DE genes when assessing individual desiccation times (Figure 3). In total, 303 genes were DE in both strains, where 150 and 110 genes were up- and down-regulated, respectively, in both strains with an additional 43 genes being significantly upregulated in Lm 08-5578, but significantly downregulated in Lm 568 (Figure 5). Interestingly, none of the communal DE genes were found to be upregulated in Lm 568 while being downregulated in Lm 08-5578.

**FIGURE 6:**
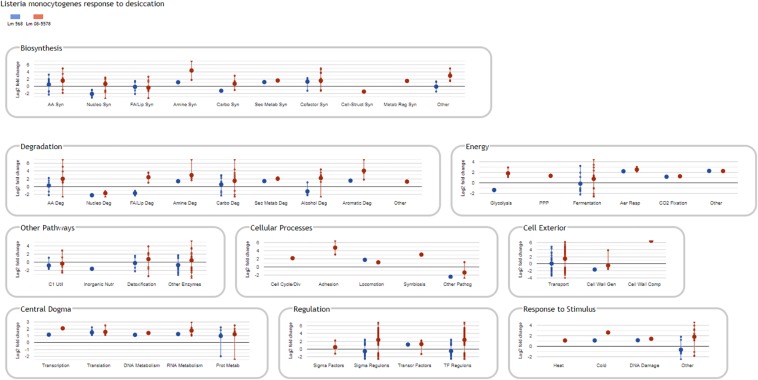
BioCyc Omics Dashboard showing significant changes in *L. monocytogenes* gene expression in response to desiccation stress. Only DE genes (LFC > 1, adjusted *p-*value < 0.05) in response to desiccation in either Lm 568 (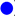) or Lm 08-5578 (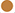) are included in the different biological panels and subsystems. Large dots show the average regulation of gene(s) in each biological subsystem, whereas small dots are individual gene values connected by a vertical line. The vertical lines link the maximum regulated genes and are not standard deviations. As a result of averaging values, the average Log_2_ fold change in a subsystem can be zero.

**FIGURE 7:**
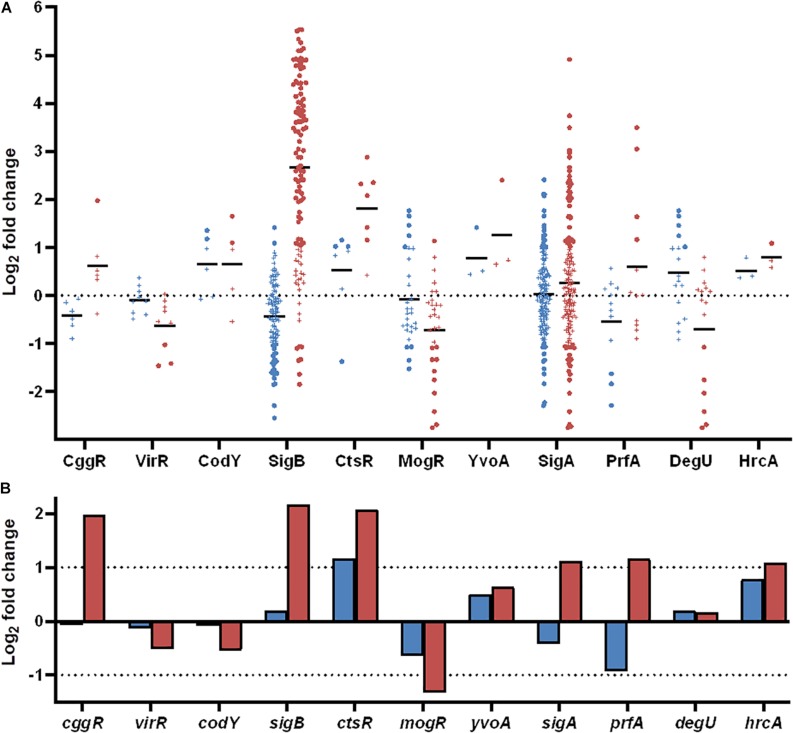
Differentially expressed genes during desiccation of *L. monocytogenes* 568 and 08-5578 grouped according to regulons and transcription factors as annotated in the BioCyc database. **(A)** Significantly up- or downregulated (LFC > 1, adjusted *p*-value < 0.05) genes in response to desiccation in either Lm 568 (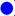) or Lm 08-5578 (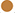) are presented in their respective regulons as dots whereas genes below significance cut-off are indicated by (+). Means (—) are the average regulation of genes in each regulon. **(B)** Log_2_ fold change of transcription factors in response to desiccation in Lm 568 (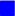) or Lm 08-5578 (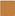).

Information about strain DE genes was transferred to the BioCyc Omics Dashboard (Paley et al., 2017) to visualize the differences between the strains based on the average LFC within annotated cellular systems and subsystems (Figure 6). Generally, desiccation stress caused elevated expression of metabolic subsystems in both strains, including increased amino acid and cofactor synthesis, degradation of amino acids, amines, carbohydrates, and aromatic compounds as well as energy metabolism. The distribution of upregulated genes within metabolic systems is supported by observations from the study of Jarvis et al. (2017), which used metabolic inhibitors on starving *L. monocytogenes* to show that they retained an active metabolism and were not in a dormant state. The panel of central dogma activities including transcription, translation, as well as metabolism of DNA, RNA and proteins showed increases in response to desiccation stress (Figure 6). The BioCyc database is not fully annotated in regards to all stress and stimulus responses, but those identified (heat, cold, DNA damage, and detoxification) were all upregulated. The enhanced desiccation stress response in Lm 08-5578 was equally evident in the presented biological subsystems (Figure 6). A look at the LFCs of DE genes related to the regulation subsystems (i.e., sigma factors and regulons, transcription factors, and regulons) showed that Lm 08-5578 contained a high number of upregulated genes, while the average changes in Lm 568 were close to zero. These differences may explain the larger transcriptomic response to desiccation observed in Lm 08-5578 compared to Lm 568. For example, the gene for the alternative sigma factor σ^B^ (*sigB*) was 4.5-fold upregulated in Lm 08-5578, while the expression in Lm 568 stayed constant in all wet and desiccated samples at a 2.5-fold lower transcription level compared to desiccated Lm 08-5578 cells (Supplementary Table S1). σ^B^ positively regulates at least 288 genes (Chaturongakul et al., 2011) and has a major role in initiating the general stress response when *L. monocytogenes* faces environmental stresses such as osmotic (Becker et al., 1998; Fraser et al., 2003), starvation (Chaturongakul and Boor, 2004) pH, oxidative, and high hydrostatic pressure (Ferreira et al., 2001; Wemekamp-Kamphuis et al., 2004b). Cabrita et al. (2015) have previously shown how *sigB* transcription was higher in a persistent strain compared to a presumed non-persistent strain. The role of σ^B^ in desiccation tolerance is less known. Huang et al. (2015) observed decreased desiccation resistance of a *sigB* Lm 568 null mutant in nutrient-limited peptone saline and minimal media substrates, but found no differences between the mutant and the wild-type during desiccation in rich media. This points to Lm 568 only needing *sigB* (and its regulon) for survival during desiccation stress under nutrient limited conditions as opposed to nutrient-rich conditions. The upregulation of *sigB* in Lm 08-5578 led to members of the σ^B^-regulon being overrepresented (*p* = 2.53 × 10^–16^) during desiccation with significantly upregulation of at least 85 σ^B^-regulon members that were not upregulated in Lm 568 (Figure 7A). Examples of upregulated genes in the present study (Supplementary Table S2) include the carnitine osmolyte uptake system *opuCABCD* (Sue et al., 2004), pyrimidine biosynthesis genes *pyrABCDEFP* (Liu et al., 2017b), carbohydrate metabolism genes with the PTS-mannose operon *mpoABCD*, universal stress proteins *uspA*, central glycolytic genes regulator *cggR*, stress response protein *csbD*, general stress protein 26 (*lmo2748*), Listeria adhesion protein *lapB* and succinate-semialdehyde dehydrogenase *gabD* (Orsi et al., 2015).

### Oxidative Stress Response Mechanisms in Desiccated *L. monocytogenes*

Removal of water from the cell surface of desiccating *L. monocytogenes* led to increased expression of genes related to oxidative stress including several known members of the σ^B^-regulon. Among these were the superoxide dismutase encoding gene, *sod* (Liu et al., 2017b), which was threefold and fourfold upregulated in Lm 568 and Lm 08-5578, respectively (Supplementary Table S2). Superoxide dismutase (Sod) protects *L. monocytogenes* from reactive oxygen species (ROS) encountered during oxidative stress and is important for virulence with Δ*sod* mutants having impaired intracellular survival (Archambaud et al., 2006). Desiccation also caused both strains to upregulate expression of the *mntABCH* operon (Table 2) to promote import of manganese, which is the co-factor for Sod (Vasconcelos and Deneer, 1994). Accumulation of manganese is crucial not only as cofactor of superoxide dismutase, but can also supplement iron(II) to render mononuclear enzymes less sensitive to oxidation (Anjem and Imlay, 2012). Along with *sod*, catalase (*kat)* was upregulated in both Lm 568 (2.3-fold) and Lm 08-5578 (4.9-fold). Catalase plays a crucial role in the oxidative stress response against ROS by converting the cell toxic H_2_O_2_ formed by Sod into water and oxygen (Mongkolsuk and Helmann, 2002). Also, threefold upregulated in both strains were genes encoding thioredoxin (*trxA*), thioredoxin reductase (*trxB*), and thiol peroxidase (*tpx*), which are involved in detoxification of the cell during oxidative stress and maintenance of reducing environments in the cytoplasm (Holmgren, 1985). *L. monocytogenes* possesses another hypothetical thioredoxin reductase (*lmo2390*), which was upregulated (3.3-fold) in both strains (Supplementary Table S2). Furthermore, three thioredoxins were also upregulated in response to desiccation: *lmo2830* in both strains, *lmo2424* in Lm 568 only and *lmo1609* in Lm 08-5578 only, while the two remaining thioredoxins (*lmo1903* and *lmo1903*) were not DE in any of the strains. In contrast to the oxidative stress genes above, *recA*, encoding a DNA repairing recombinase A (Imlay, 2003), were not DE expressed, but showed consistent high levels of expression in both wet and desiccated samples. The quinol oxidase aa_3_ operon *qoxABCD* was part of the core response to desiccation (Table 2) and recently confirmed as part of the σ^B^-regulon (Liu et al., 2017b). The *qoxABCD* operon was upregulated (>fourfold) in both strains with higher fold changes in Lm 08-5578. Interestingly, levels of QoxA was reported to be 50 times more abundant in the exoproteome of a persistent *L. monocytogenes* strain 6179 compared to the more stress sensitive EGD-e (Rychli et al., 2016). *qoxABCD* is also regulated by the primary householding sigma factor σ^A^ (*sigA*). *sigA* were not DE in neither strains, but *sigA* transcription levels increased almost twofold in Lm 08-5578 and members of the σ^A^-regulon (*sigA*) was found to be significantly (*p* = 4.24 × 10^–14^) overrepresented during desiccation of this strain (Figure 7A). Recently, Boonmee et al. (2019) showed how σ^A^ in a Δ*sigBCHL* mutant could compensate for the lack of alternative sigma factors resulting in survival of the mutant during exposure to bile not being different from survival of the wild type. This compensatory effect of σ^A^ may explain the behavior of Lm 568 during desiccation where *sigB* was not upregulated during desiccation (this study) and its Δ*sigB* mutant exhibited similar survival when desiccated in a nutrient rich medium (Huang et al., 2015). It may also be that the transcription level of *sigB* in the wet control samples of Lm 568 was at a sufficiently high level to support a stress response throughout 48-h of desiccation.

### Expression Changes in Regulatory and Mobile Elements

Where some of the differences in DE between the strains can be explained by the effect of the σ^B^ regulon (Figure 7A), as discussed above, σ^B^ is only one of several alternative sigma factors (i.e., σ^C^, σ^H^, σ^L^) in *L. monocytogenes* that together with the housekeeping σ^A^ and other transcription factors regulate a wide range of functions by transcription control (Chaturongakul et al., 2011). In both strains, the sigma factor C (*sigC*) was twofold downregulated with expression levels of *sigC* being ten times lower than *sigB* (Supplementary Figure S3). The *sigH* and *sigL* alternative sigma factors were significantly (adjusted *p*-value < 0.05) downregulated in Lm 08-5578, but below the twofold threshold, and also expressed at a low level compared to other transcription factors (Supplementary Figure S3). These findings are supported by transcriptomic and phenotypic analyses of Δ*sigC*, Δ*sigH* and Δ*sigL* mutants, who exhibited limited changes in their phenotypes, whereas the Δ*sigB* exhibited multiple defects (Chaturongakul et al., 2011). The virulence regulator *prfA* was significantly (adjusted *p*-value < 0.05) upregulated (threefold) in Lm 08-5578 (Figure 7B). Three PrfA-regulon members, *inlA* (internalin A, twofold), *bsh* (bile salt hydrolase, 11-fold), *lapB* (Listeria adhesion protein B, eightfold), became upregulated during desiccation (Supplementary Table S2). These genes are also positively regulated by σ^B^, as is *prfA* itself (Ribeiro et al., 2014). As 10 other genes, which are positively regulated by *prfA* (Chaturongakul et al., 2011), showed no DE in response to desiccation in Lm 08-5578, it may be that upregulation of *prfA*, *inlA*, *bsh*, and *lapB* was linked to the upregulation of *sigB* (Figure 7A). This correlates with observations for Lm 568 where the lack of *sigB* upregulation and downregulation (1.9-fold) of *prfA* (Figure 7B) may have led to the detected downregulation of *inlA* (fourfold), *inlB* (threefold), and *bsh* (fivefold) (Supplementary Table S2).

Lm 08-5578 carries a five gene small stress survival islet (SSI-1), which is composed of genes *lmo0444-lmo448* encoding a hypothetical protein, a transcriptional regulator, a penicillin acylase, a glutamate decarboxylase and a glutamate antiporter (Ryan et al., 2010). In response to desiccation, *lmo0445* the transcriptional regulator of SSI-1 was significantly upregulated (eightfold), while the *lmo0446 and lmo0447* were twofold upregulated (Supplementary Table S2). The upregulation of SSI-1 in Lm 08-5578 may be related to the SSI-1 being induced by σ^B^ (Raengpradub et al., 2008; Ryan et al., 2010). Hingston et al. (2017b) found in their comparison of 166 *L. monocytogenes* strains representing many clonal complexes that possession of SSI-1 did not increase tolerance to desiccation, salt, cold or acid stress. This is in contrast to mutagenesis studies which comparing isogenic wild-type and mutants showed SSI-1 to enhance tolerance to cold, acid and salt stress (Cotter et al., 2005; Ryan et al., 2010). Lm 08-5578 also harbors pLm5578, which is a 77 kbp plasmid comprised of 79 genes (Gilmour et al., 2010). Among the predicted pLm5578 genes are: *proW*, an osmolyte transporter, a ClpL chaperone and a NADH peroxidase, which were all upregulated in response to 6% NaCl (Hingston et al., 2019) indicating a role for the plasmid during osmotic stress. Interestingly, none of the pLm5578 genes were DE (LFC < 1, adjusted *p-*value < 0.05) during desiccation of Lm 08-5578, meaning that this plasmid may be redundant in the strain’s desiccation stress response.

### Differential Expression of Osmotic Stress Genes in Response to Desiccation Stress

Desiccated cells experience a stress related to osmotic stress (Billi and Potts, 2002), which bacterial cells can counter by accumulation of compatible solutes (osmolytes or osmoprotectants) such as proline, carnitine and glycine betaine to help restore and maintain turgor pressure (Wemekamp-Kamphuis et al., 2004a). Drying Lm 568 and Lm 08-5578 significantly upregulated (1.5–2 fold) the glycine betaine transporter operon *gbuABC*. In both strains there were no changes in expression of the secondary glycine betaine *betL* (Supplementary Table S1), but expression of the asRNA of *betL* was lower in both strains during desiccation and significantly downregulated in Lm 08-5578 potentially leading to increased translation of BetL. In contrast, the σ^B^-regulated carnitine transport system *opuCABCD* (Fraser et al., 2003) were highly upregulated (>27-fold) in Lm 08-5578 while being unchanged in Lm 568 likely due to lack of *sigB* upregulation. Proline biosynthesis coding genes *proABC* were not DE, despite a high upregulation (*lmo1738-40*) of the relevant precursor glutamine transporter system (Table 2). TSB-glu contains glycine betaine, carnitine, and proline. Growth in defined minimal media with these osmolytes (1 mM) prior to desiccation has been shown to increase survival of Lm 568, while presence of osmolytes in the minimal media exerted a modest protective effect on Lm 568 being desiccated (Huang et al., 2015). Together these observations indicate that osmolytes play a role in the initial response of *L. monocytogenes* to desiccation.

The osmotic stress sensing two component system of *lisRK* (Sleator and Hill, 2005) was twofold downregulated in Lm 568, while the LisR and σ^B^ regulated heat-shock chaperone HtrA (*htrA)* threefold was upregulated in Lm 08-5578. Several other chaperones have been related to the osmotic stress response in *L. monocytogenes* including *htrA*, *hfq*, *dnaK*, *clpC*, *clpP*, *cspA*, and *cspD* (Soni et al., 2011; Burgess et al., 2016). Of these *cspA* were upregulated in both strains. Lm 08-5578 additionally upregulated groE, *hfq*, *htrA* clpC, and *clpP*. The cold-shock protein A encoding *cspA* gene was threefold upregulated in both strains to become the most expressed gene during desiccation (Supplementary Table S1). Cold shock proteins (CspABD) are needed for growth at low temperatures and known to contribute to osmotic stress resistance in *L. monocytogenes* (Schmid et al., 2009), however, as opposed to CspA, the genes of CspB and CspD were not DE during desiccation of the bacterium in the present study.

### Desiccation Decreases Expression of Genes Involved in Motility and Chemotaxis

Analysis of 44 motility and motility related chemotaxis genes (*lmo0675-lmo0718* and *lmo1699*) identified significant downregulation (>twofold, adjusted *p-*value < 0.05) of motility genes in both strains including the two component chemotaxis system *cheA/cheY* (Table 3), as well as *lmo0703-704*, *lmo0694*. Moreover, Lm 568 downregulated *fliY* (*lmo0693*, flagellar motor switch protein), while Lm 08-5578 downregulated the chemotaxis protein *lmo1699* (Supplementary Table S2). A study by Cheng et al. (2018) showed how in the absence of either FlhB, FliM or FliY *L. monocytogenes* completely lost its ability to produce flagella. Downregulation of motility genes in response to desiccation have also been observed in *Pseudomonas putida* (Van de Mortel and Halverson, 2004) and *Enterobacter sakazakii* (Riedel and Lehner, 2007). Lm 08-5578 downregulated six additional flagellar biosynthesis proteins [*fliNPQR* (*lmo0675-0678*) and (*flhAB* (*lmo0679-0680*)], while flagellar motor rotation proteins encoding genes (*motAB*) were significantly upregulated in Lm 568 (Supplementary Table S2). These downregulated flagella biosynthesis genes *lmo0675-0680* are the first genes in the flagellar operon and negatively controlled by MogR, σ^B^, σ^L^, and σ^H^, and presumably also negatively regulated by a long antisense transcript (lasRNA) anti0677, while positively regulated by CtsR (Toledo-Arana et al., 2009; Chaturongakul et al., 2011). While *L. monocytogenes* is highly flagellated and motile at temperatures 30°C and below, it downregulates motility genes including *flaA* (flagellar protein) above 30°C with limited expression at 37°C (Peel et al., 1988). Differences in motility have been used to differentiate the capacity to adapt to cold temperatures in *L. monocytogenes* strains with less motile strains adapting faster than more motile strains with microarray data correlating these findings to expression of motility genes (Cordero et al., 2016). Similarly, Cabrita et al. (2015) saw significant differences in relative gene expression of *flaA* between a persistent and presumed non-persistent strain, with increased expression in the non-persistent strains. Interestingly, *sigB* expression was lowest in the non-persistent strain. These findings are similar to the present study where Lm 568 exhibited lower expression of *sigB* and less downregulation of motility genes during desiccation as compared to the Lm 08-5578 outbreak strain (Supplementary Tables S1, S2). In fact, Lm 08-5578 reduced its expression of 28 motility related genes (*cheARVY*, *fliDEFIMPQRY*, *flgBCDGKL*, *flhABF*, *motAB*, *flaA*, *lmo0698*, *lmo0708*, and *lmo1699*) to significantly (*p* < 0.05) lower levels compared to Lm 568 (Supplementary Figure S4). The gene encoding the motility repressor (*mogR*) was downregulated (twofold, Figure 7A). MogR is supposed to prevent expression of motility genes including *flaA* (Grundling et al., 2004) meaning that lowered levels of *mogR* should increase levels of motility genes. However, the effect of *mogR* regulation may have been overruled by other regulators including σ^B^ as suggested by Chaturongakul et al. (2011). That motility plays a major role in tolerance to desiccation was also reported in the study of insertional mutants in Lm 568, which identified seven desiccation tolerant immotile mutants with insertions in flagella associated genes (Hingston et al., 2015). The transcriptomic data from the present study (Supplementary Table S1) revealed that except for *motB* both strains downregulated the genes (*fliP*, *flhB*, *flgD*, *fliM*, *fliY*, *flgL*), which had been interrupted in the insertional mutagenesis study. It appears that a lack of motility or decreased motility is advantageous to survival during desiccation possibly by reducing the energy going to these processes as suggested by Hingston et al. (2015).

### Anti-sense Transcription in Desiccation Stressed *L. monocytogenes*

Transcriptomes from Lm 568 and Lm 08-5578 contained antisense RNA (asRNA) levels of >10 transcripts per kilobase million (TPM) for 61 and 71%, respectively, of the ORFs. However, asRNA transcript levels only surpassed >100 TPM for 194 and 170 genes in Lm 568 and Lm 08-5578, respectively (Supplementary Table S1). In comparison, more than 1900 mRNA transcripts were found at levels of >100 TPM in samples from both strains. These results are in line with previous findings for *Bacillus anthracis* and *L. monocytogenes*, where ∼30% of the ORFs showed no or minimal expression of asRNA in *B. anthracis* and only 56 asRNA transcripts showing higher levels of expression in *L. monocytogenes* (>1000 PE reads) (Passalacqua et al., 2012; Hingston et al., 2017a). In response to desiccation, 17 and 69 asRNA transcripts were significantly upregulated throughout the desiccation in Lm 568 and Lm 08-5578, respectively, while 14 and 83 asRNA transcripts were downregulated (Supplementary Table S3). Shared among the strains were 11 upregulated and 9 downregulated asRNA transcripts, of which the asRNA of *lmo1846* (*anti1846*) was the most upregulated (>10 fold) and expressed during desiccation (Supplementary Table S3). *anti1846* is a long antisense transcript (lasRNA) overlapping several genes and has been suggested to work as an excludon, that inhibits *lmo1846* while serving as coding sequence of another operon (*lmo1843*-*45*) (Wurtzel et al., 2012). Interestingly, *anti1846* is adjacent to the *mntABC* operon (*lmo1847-49*) of the manganese transport complex identified as an essential part of the upregulated response to desiccation in this study, while expression of *lmo1846* encoding an efflux pump was significantly downregulated. It is, however, not known if *anti1846* has any effect on the *mntABC* operon. Several other lasRNAs were upregulated in Lm 08-5578, including *anti0936* (>22 fold) whose target mRNA *lmo0936* was downregulated during desiccation. Identification of *anti0936* and its downregulation of *lmo0936* was reported by Wehner et al. (2014) to be part of the bacterium’s response to intracellular growth. In addition the 5.8 kb long *anti0605*, a σ^B^ induced excludon, which limits transcription of the multidrug efflux pump *lmo0605*, while serving as coding sequence of *lmo0606-08* (Wurtzel et al., 2012), was upregulated (>22 fold) in Lm 08-5578. *Lmo0606-0608*, which encodes a marE transcriptional regulator and a putative ABC transporter (Wurtzel et al., 2012), was upregulated in both Lm 568 and Lm 08-5578 (Supplementary Table S2). *Lmo0607* is positively regulated by σ^B^ (Raengpradub et al., 2008), and transporter complex of *lmo0607-8* was upregulated in response to intercellular growth (Schultze et al., 2015).

Moreover, a lasRNA covering three genes required for flagellum synthesis *fliN* (sixfold), *fliP* (12-fold), and *fliQ* (14-fold) were upregulated in Lm 08-5578 correlating with these genes also being the most downregulated motility genes (fivefold decrease) during desiccation (Supplementary Table S2). The asRNA expression levels of *fliNPQ* were 10-66 × higher than their corresponding mRNAs during desiccation (Supplementary Table S1). *fliNPQ* makes up the first three genes of the big *mogR* regulated motility operon with *fliNPQRB-flhAF-flgG-cheR-motAB-gmaR-cheV* (Orsi et al., 2015). lasRNA of *fliNPQ* was identified as the flagellum biosynthesis excludon *anti0677*, a lasRNA induced by σ^B^, which overlaps and downregulates *flipNPQ* by complementation and further serves as coding sequence of *mogR* (Toledo-Arana et al., 2009; Schultze et al., 2014). σ^B^ regulation of *anti0677* transcription were confirmed by the low and unchanged levels of *anti0677* in Lm 568 being desiccated as well as lack of downregulation of *fliNPQ*, which correlates with *sigB* levels being unchanged in Lm 568 as opposed to levels in Lm 08-5578. Together these observations propose a role for several lasRNAs (*anti0605*, *anti0677*, *anti0936*, and *anti1846*) as part of the regulation of the Listerial desiccation stress response.

In both Lm 568 and Lm 08-5578 the antisense transcript of *radC* was fourfold downregulated, while the mRNA transcript levels of *radC* remained at a low level in both strains. The DNA repair protein encoding *radC* was upregulated in *L. monocytogenes 08-5923* exposed to a combination of sodium lactate and sodium acetate (Liu et al., 2017a). The asRNA transcripts of the glycine-betaine transporter *betL* and internalin B (*inlB*) were significantly (threefold, adjusted *p*-value < 0.05) downregulated in Lm 08-5578 to putatively increase translation of BetL and InlB during desiccation. In contrast, Lm 08-5578 highly upregulated, by 124-fold, *lmo0913* (encoding succinate semialdehyde dehydrogenase) whose asRNA was also upregulated (fivefold). This could be explained by a feedback mechanism or asRNA working in a fashion that stabilizes mRNA transcripts of *lmo0913* as reported by Schultze et al. (2014).

## Conclusion

In this study, we present the first time-course RNA-seq study of desiccating *L. monocytogenes* under industrially relevant conditions to simulate a weekend shut-down (48 h) in a food processing plant. The use of two different *L. monocytogenes* strains allowed us to identify a common core (i.e., strain independent) response of significantly up- or downregulated genes during the 48-h desiccation period, which resulted in a ∼2 Log reduction in survivors. Among significantly upregulated genes were energy, osmotic and oxidative stress related genes, with the oxidative stress being the most upregulated stress response. Both strains also responded to desiccation by downregulating genes involved in anaerobic and cold growth as well as motility. Significant strain differences were also detected, where the food outbreak strain Lm 08-5578 DE 1.9 × more genes (including the *sigB* regulon) compared to Lm 568 during desiccation. Also, the upregulation of several antisense transcripts was observed during desiccation including ones regulating flagellum biosynthesis and motility genes. Lastly, it was revealed that *L. monocytogenes* adapted to desiccation stress within the first 6 h.

## Data Availability Statement

FastQ files of HiSeq runs were deposited into the NCBI Sequence Read Archive under BioProject PRJNA578009.

## Author Contributions

MK and LT conceived the study, designed the laboratory work, performed RNA-seq analysis, interpreted the analyzed data, and co-wrote the manuscript. MK performed the laboratory work.

## Conflict of Interest

The authors declare that the research was conducted in the absence of any commercial or financial relationships that could be construed as a potential conflict of interest.

## Footnotes

1 https://biocyc.org/

2 http://www.pantherdb.org/

3 https://cit.kuleuven.be/biotec/software
