# Supplementary material for: Initial Transcriptomic Response and Adaption of Listeria monocytogenes to Desiccation on Food Grade Stainless Steel
Source: Front Microbiol. 2020 Jan 22;10:3132. doi: 10.3389/fmicb.2019.03132 (PMC6987299; doi:10.3389/fmicb.2019.03132)
Supplement: Supplementary file 1 [file Image_1.pdf]

Supplementary Material

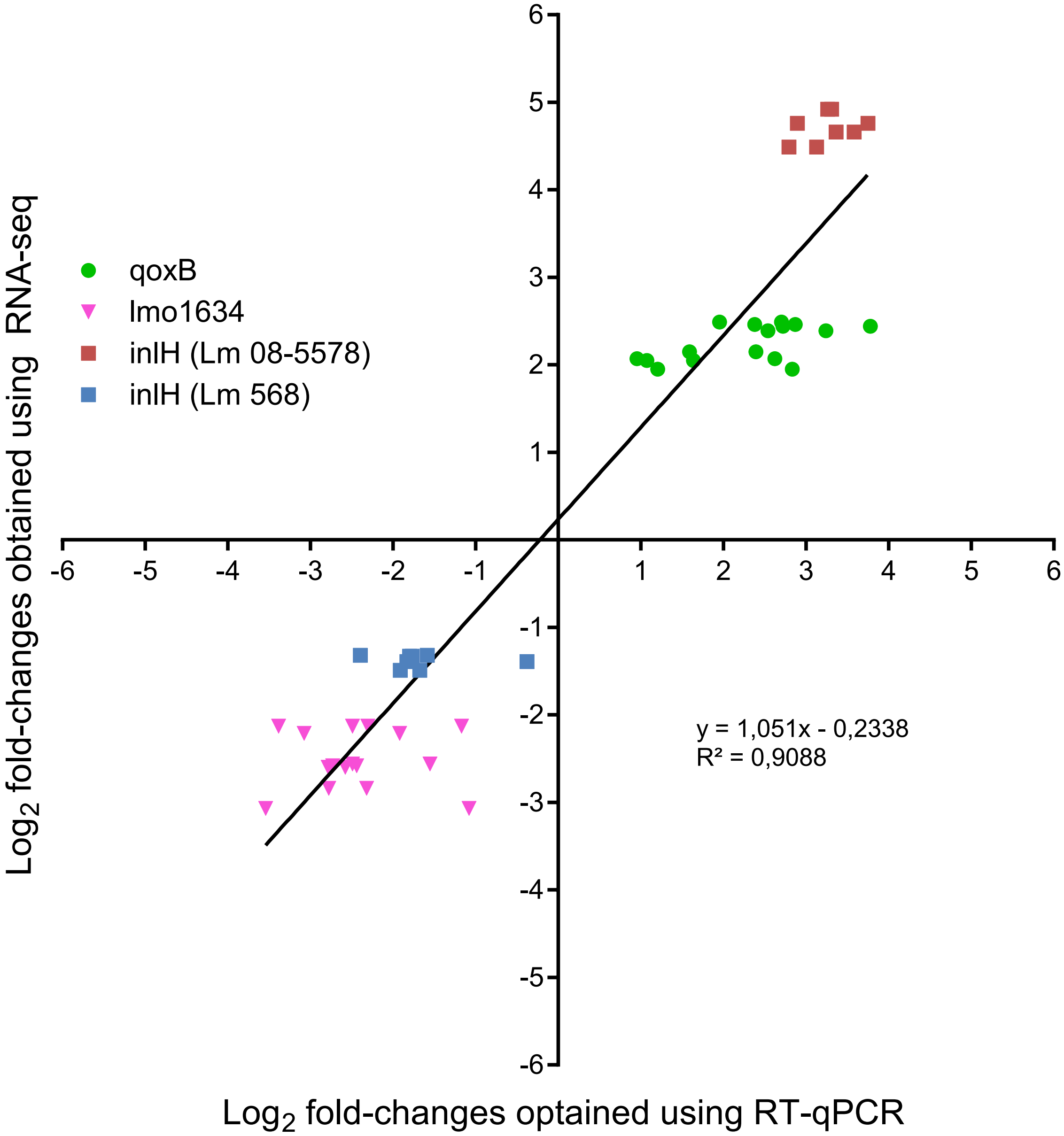

Supplementary Figure 1. Correlation between $\log_2$ fold-changes obtained using RNA-seq and RT-qPCR. Points represent the differential expression values for genes: *qoxB* (●), *lmo1634* (▼) and *inlH* in Lm 08-5578 (■) and Lm 568 (■). The x-axis represents the levels obtained using RT-qPCR while the y-axis represents the differential expression levels obtained using RNA-seq. RT-qPCR were performed on twenty different RNA-samples from experiment H and P in technical qPCR duplicates.
