# Supplementary material for: Initial Transcriptomic Response and Adaption of Listeria monocytogenes to Desiccation on Food Grade Stainless Steel
Source: Front Microbiol. 2020 Jan 22;10:3132. doi: 10.3389/fmicb.2019.03132 (PMC6987299; doi:10.3389/fmicb.2019.03132)
Supplement: Supplementary file 2 [file Image_2.pdf]

frontiers

Supplementary Material

	0 hours	6 hours	12 hours	24 hours	48 hours	Lm 08-5578
0 hours	0.91	0.76	0.73	0.71	0.78	0 hours
6 hours	0.74	0.92	0.99	0.99	0.99	6 hours
12 hours	0.76	0.98	0.91	0.99	0.98	12 hours
24 hours	0.74	0.99	0.99	0.90	0.97	24 hours
48 hours	0.78	0.97	0.99	0.99	0.93	48 hours
Lm 568	0 hours	6 hours	12 hours	24 hours	48 hours	

Supplementary Figure 2. Correlation coefficient (R^2) values between transcriptomes. Transcriptomic correlations are based on the normalized (TPM) gene counts for each time point as presented in Table S2 composed of 3055 open reading frames. Values with blue shading are correlations between transcriptomes of Lm 568, while values with red shading are correlations between transcriptomes of Lm 08-5578. Values with white shading are correlations between transcriptomes of the two strains Lm 568 and Lm 08-5578 before and during desiccation. Each transcriptome is based on the average values from two biological replicates as experiment N was excluded from this analysis.
