# Supplementary material for: Initial Transcriptomic Response and Adaption of Listeria monocytogenes to Desiccation on Food Grade Stainless Steel
Source: Front Microbiol. 2020 Jan 22;10:3132. doi: 10.3389/fmicb.2019.03132 (PMC6987299; doi:10.3389/fmicb.2019.03132)
Supplement: Supplementary file 3 [file Image_3.pdf]

Supplementary Material

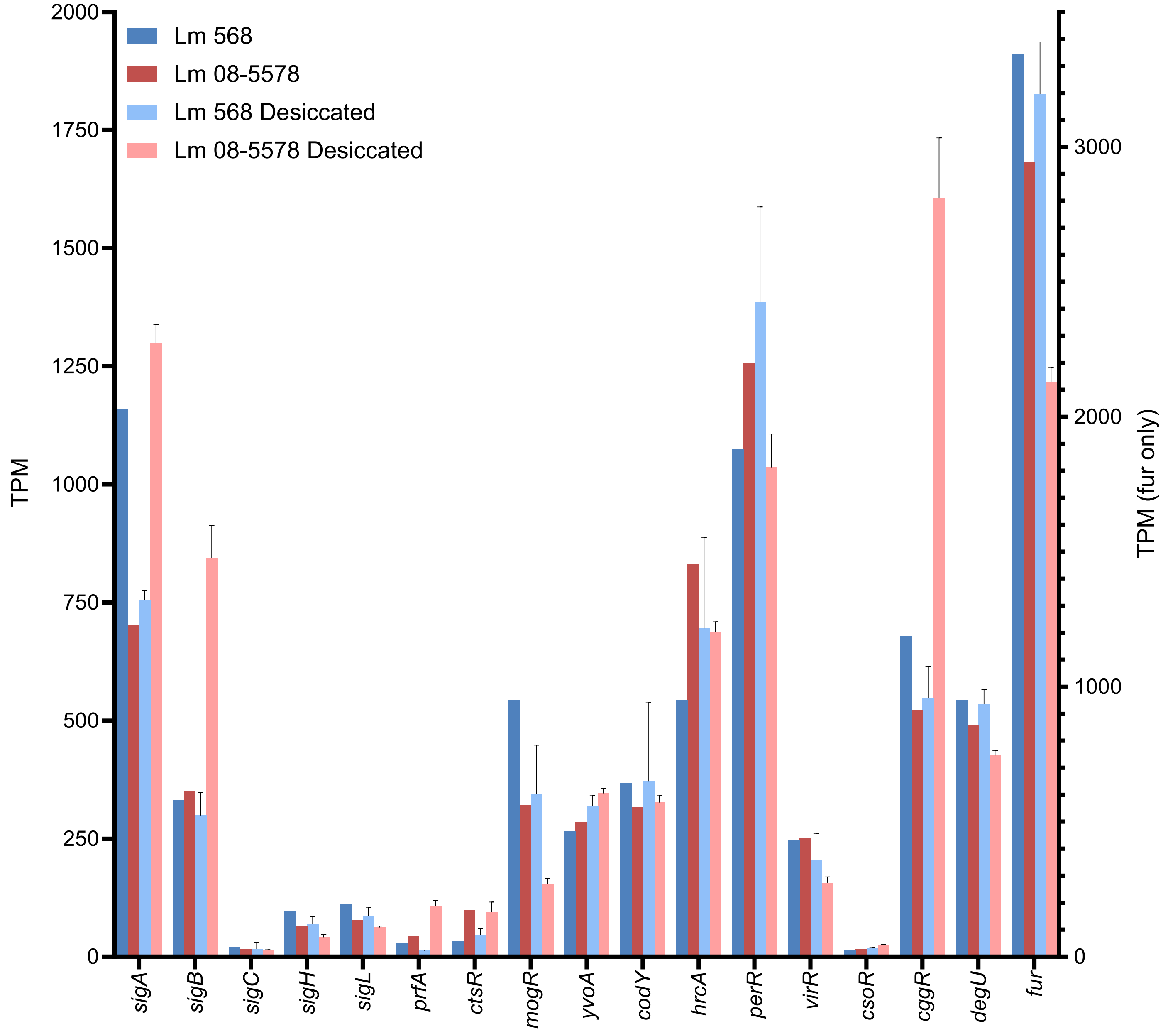

Supplementary Figure 3. Transcript levels of major transcription factors in *L. monocytogenes* in wet and desiccated cells. Transcription level is based on normalized gene counts (Transcript per kilobase million (TPM)) from either wet cells (n=2) or desiccated samples (n=8) in either Lm 568 (■) or Lm 08-5578 (■). The *fur* gene is plotted separately on the right y-axis due to high counts.
