# Supplementary material for: Initial Transcriptomic Response and Adaption of Listeria monocytogenes to Desiccation on Food Grade Stainless Steel
Source: Front Microbiol. 2020 Jan 22;10:3132. doi: 10.3389/fmicb.2019.03132 (PMC6987299; doi:10.3389/fmicb.2019.03132)
Supplement: Supplementary file 4 [file Image_4.pdf]

Supplementary Material

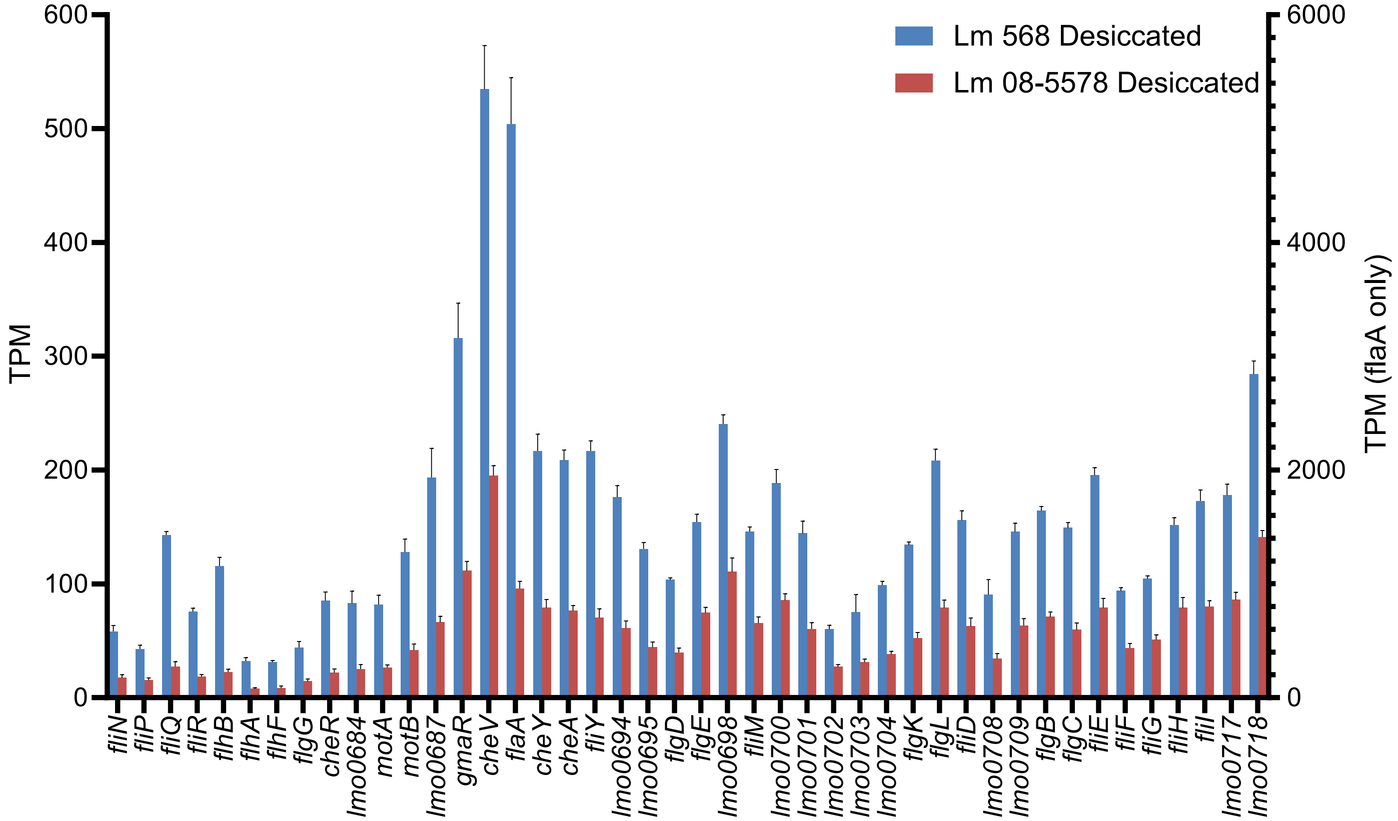

Supplementary Figure 4. Transcript levels of motility and chemotaxis genes in desiccated *L. monocytogenes* 568 and 08-5578. Transcription levels are based on normalized gene counts (Transcript per kilobase million (TPM)) from eight desiccated samples (n=8) in either Lm 568 (■) or Lm 08-5578 (■). The flagella coding gene *flaA* is plotted separately on the right y-axis due to very high counts.
